# Folic acid and breast cancer risk

**DOI:** 10.1186/1897-4287-10-S4-A7

**Published:** 2012-12-10

**Authors:** Katarzyna Durda, Katrzyna Jaworska-Bieniek, Krzysztof Kąklewski, Jan Lubiński, Anna Jakubowska

**Affiliations:** 1 International Hereditary Cancer Centre, Department of Genetics and Pathology, Pomeranian Medical University, Szczecin, Poland; 2 Postgraduate School of Molecular Medicine, Warsaw Medical University, Warsaw, Poland

## 

Recent studies indicate that the selected micro-and macro-elements and vitamins may significantly influence the risk of cancer. Folic acid is a vitamin B which plays an important role in several processes in organism. Folates are important cofactors in the transfer and utilization of one-carbon-groups and play a key role in the remethylation of methionine thus providing essential methyl groups for numerous biological reactions. Furthermore, folates donate one-carbon units in the process of DNA-biosynthesis with implications for the regulation of gene expression, transcription, chromatine structure, genomic repair and genomic stability. Deficiency of folic acid has been reported to be associated with numerous disorders, including heart disease, stroke and cancers.

The MTHFR gene produces a key enzyme in folate metabolism which catalyses the reactions essential for nucleotide biosynthesis and DNA methylation.

The aim of study was to analyze an association of folic acid concentrations and genetic variants in the MTHFR gene with breast cancer risk in patients with BRCA1 mutation.

Study group consisted of 155 breast cancer patients and 155 healthy women from the paired control group matched to cases by year of birth, cancer family history, adnexectomy, smoking. From all individuals blood sample was collected and from cancer cases was taken before treatment. Folic acid concentration was quantitatively measured in blood plasma by HPLC chromatography (Flexar HPLC, Perkin Elmer). Two functional SNPs in the MTHFR gene, 677 C>T (rs1801133) and 1298 A>C (rs1801131), both associated with reduced enzyme activity, have been tested by TaqMan on LightCycler 480.(Roche Diagnostic). Individuals were divided into four quartiles depending on folic acid concentration and number of cases and controls in each quartile was compared. Analysis was made depending on menopausal status defined as ≤ 50 and >50 years old.

In a group of >50 years old, individuals classified in the first quartile (<17,35μmol/l) had a lower risk of breast cancer than patients with higher folate level. Whereas in a group of ≤50 years old, individuals classified in the 3 quartile (24,35 – 31,88 μmol/l ) had a significantly lower risk of breast cancer than those with folate level between 32,02 – 54,42 μmol/l. Analysis of correlation between the level of folic acid and genetic variants 677 C/T and 1298 A/C in the MTHFR gene performed in 2 groups (>50 and ≤50 years) revealed:

- for carriers of 1298 nCC and ≤50 years old significantly lower risk of breast cancer in individuals classified between 16,6-31,88 μmol/l in comparison to patients with lower and higher folate level.

- for carriers of 677 CC and ≤50 years old lower risk of breast cancer in individuals classified between 19,86-32,02 μmol/l than patients with lower and higher folate level.

- for carriers of 677nCC and ≤50 years old significantly lower risk of breast cancer in individuals classified between 5,94-14,8 μmol/l in comparison to patients with higher folate level.

- for carriers of 1298 AA and >50 years old lower risk of breast cancer in individuals classified in the first quartile (<18,6μmol/l) in comparison to patients with higher folate level.

- for carriers of 1298 nCC and >50 years old lower risk of breast cancer in individuals classified in the first quartile (<17,11μmol/l) in comparison to patients with higher folate level.

- for carriers of 677 CC and >50 years old lower risk of breast cancer in individuals classified in the first quartile (<18,62μmol/l) in comparison to patients with higher folate level.

- for carriers of 677 nCC and >50 years old lower risk of breast cancer in individuals classified in the first quartile (<17,1μmol/l) in comparison to patients with higher folate level.

We can conclude that concentration of folic acid in the range of 18 - 32 μmol/l is associated with lower risk of breast cancer for women ≤50 years, with the possible exception for 677 nCC (CT+TT) where the lower risk of breast cancer is classified between 5,94-14,8 μmol/l.

Concentration of folic acid lower than 17 μmol/l is associated with lower risk of breast cancer for women >50 years.

